# Health and Well-Being of Athletes During the Coronavirus Pandemic: A Scoping Review

**DOI:** 10.3389/fpubh.2021.641392

**Published:** 2021-04-16

**Authors:** Raven Haan, Mariyam Essa Ali Alblooshi, Dawood Hasan Syed, Khaled Khalifa Dougman, Hashel Al Tunaiji, Luciana Aparecida Campos, Ovidiu Constantin Baltatu

**Affiliations:** ^1^College of Medicine & Health Sciences, Khalifa University, Abu Dhabi, United Arab Emirates; ^2^Abu Dhabi Sports Council, Abu Dhabi, United Arab Emirates; ^3^Zayed Military Hospital, Abu Dhabi, United Arab Emirates; ^4^College of Health Sciences, Abu Dhabi University, Abu Dhabi, United Arab Emirates; ^5^Center of Innovation, Technology and Education (CITE) at Sao Jose dos Campos Technology Park, São Paulo, Brazil; ^6^Institute of Biomedical Engineering at Anhembi Morumbi University - Laureate International Universities, Sao Jose dos Campos, Brazil

**Keywords:** COVID-19, SARS-CoV-2, coronavirus, athletes, psychosocial, occupational stress, primary health care

## Abstract

**Background:** The ongoing global pandemic has become the world's leading health problem, causing massive public fear and concern. Reports suggest that athletes are seeking mental health support, showing the pressures of boredom, and tension associated with their anticipated social isolation. The current study seeks to evaluate the evidence regarding the effects of the coronavirus pandemic on occupational stress in professional athletes.

**Method:** A scoping review was conducted. A comprehensive search involving Embase and PubMed databases was conducted using a combination of the following key words: COVID-19, SARS-CoV-2, coronavirus, and athletes. In this study, articles were retained if they were original studies reporting on the impact of the pandemic on professional athletes.

**Results:** Nine studies were identified as they investigated the impact of the pandemic on athletes. Most were observational and cross-sectional, and one was longitudinal. Outcome measures mainly investigated were level of depression, anxiety, and stress. Dependent variables were physical activity, nutrition, mental state, sleep quality, individual well-being, social identity, exclusivity, negative affectivity, alcohol consumption, psychological distress, and gambling habits.

**Conclusions:** This review highlights the need for proactive engagement with professional athletes, coaches, trainers, and sports councils to facilitate understanding and awareness-raising, process optimization, and delivery of consistent training and psychosocial aid and occupational therapy programs that maintain the health and well-being of athletes while minimizing occupational stress during a pandemic.

## Introduction

The coronavirus pandemic in 2020 resulted in significant global challenges. Government agencies and public health organizations have adopted public health initiatives to minimize the peak infection rate, of which the most effective recommendation known to restrict and postpone the spread of the virus is social (physical) distancing. Most countries have increasingly adopted community isolation measures to increase social distancing, such as mandatory lockdowns, isolation periods, and the closing of public areas (1). Studies investigating the impact of the coronavirus pandemic on public health have found a high level of psychological distress due to lockdown, fear of infection, and adjusting to new protective measures (2).

Social distancing measures have resulted to a substantial rise in workplace stress, with many categories of workers affected (3). Work-related factors may play a crucial role in exacerbating the effects of the pandemic isolation measures on people's mental well-being. Healthcare workers, especially those on the frontline, migrant workers, and workers in contact with the public are among the most affected with psychological critical issues in the workplace (3–5). Job insecurity and future uncertainty are significantly contributing to this “psychological pandemic” (3).

The world of sports has not been exempted from the protective measures against pandemic, with athletes unable to practice or compete or participate in international competitions. Global health recommendations have led to the cancellations and postponements of numerous athletic events in order to social distance and in an attempt to limit the spread of the virus (6). International sporting events, such as the Olympic Games, Euro (European Football Championships), and the Wimbledon Tennis Tournament, were either postponed or finally canceled in 2020. In addition to adjusting to the new norms due to the pandemic, athletes in particular have undergone significant lifestyle and routine changes, interpersonal relationships, financial challenges (e.g., work loss or sponsorship), and loss of goals and satisfaction (7). Shortly after the outbreak of the coronavirus pandemic, the International Society for Sport Psychology (ISSP) released three editorials and comments on the effect of the coronavirus pandemic on athletes on the complexities and suggestions of working with athletes (8), sports psychology services (9), and tips for athletes, coaches, parents, and the sports community (10).

With limitations to practice in competitions, the potential implications to athletes are numerous, as this may affect their health with lack of training and their income as matches are canceled. In addition, there may be an element of fear of athletes catching the virus, as there have been numerous high-profile cases of professional athletes testing positive for COVID-19 (11). The fact that high-profile cases have been found positive for the disease, including professional footballers and athletes, despite their recognized high fitness levels indicates that they have not been made less vulnerable to the virus. Fear of catching this virus can lead to stress and adverse effects on the mental health of athletes during this pandemic. While everyone reacts differently to stress, and some athletes may be able to find successful coping mechanisms, other athletes may have a more pessimistic response over the uncertain period of the global pandemic that we are facing. Although anxiety is a natural response to COVID-19 and the effects it has had globally, stressors can be especially harmful to professional athletes as they can fully alter their day-to-day lives. Last but not least, athletes who have not been training due to a pandemic lockdown for some time may worry that this may have a negative effect on their sporting skills and performance. As high levels of stress can have a detrimental impact on everyday life and mental and physical health, there is a need to examine and diagnose psychological problems and deteriorating mental health among professional athletes during the COVID-19 pandemic.

The current study seeks to evaluate the evidence regarding the effects of the coronavirus pandemic on occupational stress in professional athletes.

## Methods

The scoping review was conducted according to the standards and guidelines established in the Preferred Reporting Items for Systematic Reviews and Meta-Analysis (PRISMA) with the associated extension for Scoping Reviews (12).

### Search Strategy

We conducted a systematic literature search of the Embase and PubMed databases using a combination of the following keywords: COVID-19, SARS-CoV-2, and athletes. Databases have been checked until the date of our literature searches (30 November 2020). Studies were included in this review if they were original studies focusing on the effect of the pandemic on professional athletes. In order to ensure completeness, we also checked for references to our full-text articles. References of the reported relevant reviews were also screened.

Criteria for eligibility were (1) Population: professional athletes, (2) Intervention: COVID-19 pandemic, (3) Comparator: not available, and (4) Outcomes: any qualitative or quantitative findings published in the literature related to psychological health. No restrictions were placed on study design, age, or injury type.

Exclusion criteria were studies not related to the COVID-19 pandemic, not reporting outcomes, and not in English.

### Study Selection

Three reviewers (RH, MA, DH) engaged in the database screening process and the analysis of the full text for eligibility. Disagreements between the three reviewers were resolved through dialogue and consultation with the two senior authors (LAC, OCB).

### Quality Appraisal

In line with scoping review practice, included studies have not been assessed in terms of quality (13).

## Results

**Figure 1:**
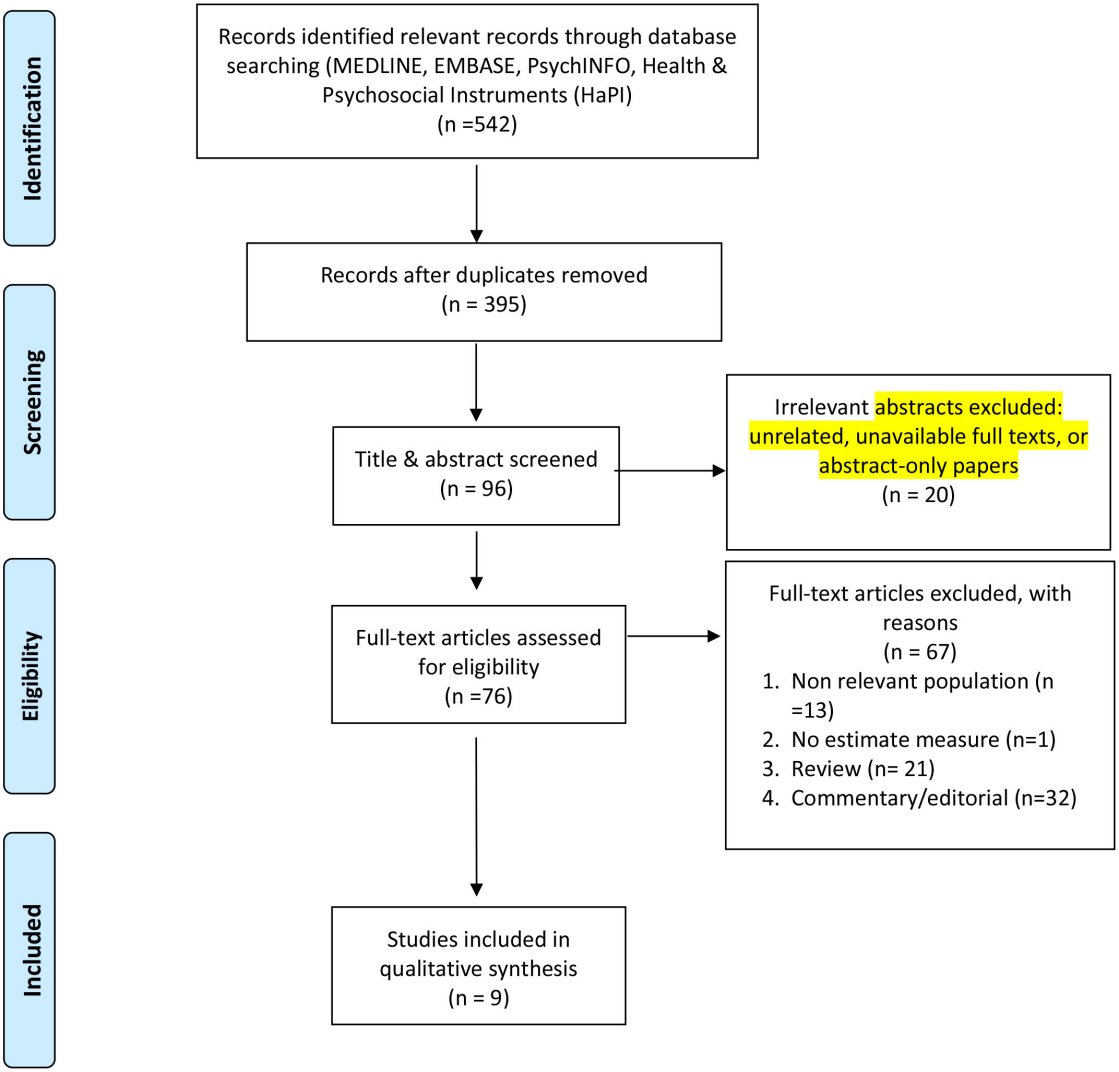
PRISMA flow diagram: original studies reporting on the impact of the coronavirus pandemic on professional athletes.

The results of the screening process are shown in the PRISMA diagram in Figure 1. Of the 542 titles and database citations imported, 395 remained after duplicates had been deleted. After title and abstract screening, 96 were suitable for full text evaluation. Of the 96 deemed suitable, 20 were excluded as unrelated, having unavailable full texts, or being abstract-only papers. Of the 76 full-text articles, a total of nine met the inclusion criteria for this scoping review.

Original research articles were cross-sectional studies (Table 1). No conflict of interest has been reported in all studies.

**Table 1:** Original studies reporting on the impact of the pandemic on professional athletes.

	**Research design**	**Participants**	**Target variables**	**Main findings**
Meyer et al. (14)	2-month prospective longitudinal cohort	1,702 male professional football players from German leagues and the officials working closely with them	(1) Onset of typical COVID-19 symptoms, (2) Positive PCR results, and (3) IgG seroconversion against SARS-CoV-2	Of the 1,702 regularly tested individuals, only eight players and four officials tested positive during one of the first rounds of PCR testing prior to the onset of team training, two players during the third round. No further positive results occurred during the remainder of the season.
Pillay et al. (15)	Cross-sectional survey	692 (67% males) athletes from South Africa	(1) Activity (2) Nutrition (3) Mental state	COVID-19 had physical, nutritional, and psychological consequences
Senişik et al. (16)	Cross-sectional survey	418 athletes of 612 volunteers from Turkey	(1) Mental health: depression and anxiety (DASS-21) and posttraumatic stress levels (IES-R) (2) Physical activity (IPAQ-SF)	Mental health status of athletes was better than the non-athlete controls. Depression levels were similar in team and individual athletes. Team athletes had a lower level of anxiety compared to individual athletes.
Costa et al. (17)	Cross-sectional survey	1,125 (45.8% men) athletes from various sports in Italy	(1) Athletic identity (AIMS scale) (2) Cognitive emotion regulation (CERQ scale)	Athletes with higher athletic identity tend to ruminate and catastrophize more.
Håkansson et al. (18)	Cross-sectional survey	1,145 (82% men) athletes in top leagues of soccer, ice hockey, and handball in Sweden	(1) Depression (2) Anxiety (3) Alcohol drinking (4) Gambling behavior (5) Problem gambling	Distress from pandemic is common in elite athletes and associated with mental health symptoms. Gambling increase during the pandemic was rare, but related to gambling problems.
McGuine et al. (19)	Cross-sectional survey	13,002 (53.1% women) USA adolescent athletes	(1) Physical activity (PFABS) (2) Anxiety (GAD-7) (3) Depression (PHQ-9) (4) Health-related quality of life (PedsQL)	Women reported a higher prevalence of moderate to severe anxiety symptoms. The prevalence of depression symptoms was highest in team sports than individual sports. The total PedsQL score was lowest (worst) for athletes from counties with the highest poverty levels.
Graupensperger et al. (20)	Longitudinal survey, before and after 1 month after university campus closures during COVID-19	234 (63% female) USA student-athletes	(1) Athletic identity change (AIMS) (2) Teammate social support (SSB) (3) Teammate social connectedness (4) Well-being (MHC-SF) (5) Depression (PROMIS) (6) COVID-specific worries	Positive correlations occurred between teammate social experiences and identity maintenance, and consequently, identity maintenance was positively correlated with psychological and social well-being and was negatively associated with depressive symptoms.
Mon-López et al. (21)	Cross-sectional survey, retrospective	187 (64.7% men) handball players from Spain	(1) Demographic variables: gender, age place of residence, number of days confined, sport level, playing position and personal experience with COVID-19 (2) Training variables: training days, training hours, intensity, and recovery. (3) Psychological variables: emotional intelligence (WLEIS-S), mood state (POMS), and resilience (BRS-II)	COVID-19 isolation had significant negative effects on the training and recovery of the athletes, as well as sleep quality, and other psychological variables.
Grazioli et al. (22)	Cross-sectional observational, after 63 days of quarantine and compared with retrospective data obtained after a regular 24-day off-season period	23 male Brazilian professional soccer players who returned to training activities after 63 days of quarantine	(1) Body composition (2) Jump and sprint performance (3) Hamstring eccentric strength (4) Intermittent cardiorespiratory fitness	Quarantine caused a substantial increase in body mass, body fat mass, 10- and 20-m sprint times, and a decrease in counter-movement jump height.

In a 2-month prospective longitudinal study at the German professional men's soccer league, coronavirus infection rate could be kept low through strict hygiene measures including regular PCR testing (14).

In South Africa, a cohort of elite and semi-elite athletes out of 15 sports reported self-reported pandemic psychological effects on physical, nutritional, and psychological health (soccer, hockey, rugby, cricket, athletics, netball, basketball, endurance running, cycling, track and field, swimming, squash, golf, tennis, karate) (15).

In Turkey, depression and anxiety symptoms were lower in athletes compared to non-athletes but were comparable in team athletes and individual athletes (16).

In Italy, professional athletes and team sports athletes demonstrated a higher athletic identity during the lockdown era (17). Cognitive emotion regulation strategies were different for gender and competitive levels. At the end of the day, athletes with a higher athletic identity appeared to ruminate and catastrophize more.

In Sweden, self-reported pandemic psychological influences have been widespread among elite soccer, ice hockey, and handball athletes, as well as worries about one's sport and one's own career linked to the crisis situation. Depression and anxiety were not significantly higher than predicted but correlated with coronavirus-related worry definitions. Fear of increased gambling during the crisis could not be explicitly shown, but at-risk gambling in male athletes was widespread and also related to a rise in pandemic gambling.

In the USA, during pandemic school closures and sport cancellations, lower levels of physical activity and quality of life and increased symptoms of anxiety and depression have been identified in female athletes, team sports players, and areas with a higher percentage of poverty (19). Due to the pandemic, student-athletes who received more social support and reported more connection with teammates reported less dissolution of their athletic identity in a survey before and 1 month after school closure (20).

In Spanish handball players, reduced training intensity, and volume were associated with reduced sleep quality and increased sleep hours during the isolation period (21).

In Brazilian soccer players, over normal off-season, 63 days of quarantine affected several physical performance tests (22).

Although results varied slightly between different sports and geographical regions, the general consensus from studies (12–17, 19, 20) is that athletes are suffering both physically and mentally from the effects of the Covid-19 pandemic and the imposed restrictions and regulations on sporting events. The results from the German prospective longitudinal study suggest that return to sporting activities may be achieved with minimal risk of infection as long as strict hygiene measures are implemented, which suggests that it may be more beneficial to athletes' physical and psychological health if they return to training and sporting events rather than self-isolate (12).

## Discussion

Studies investigating the impact of the coronavirus pandemic on public health have found a high level of psychological distress (2). Psychological distress among professional athletes and the pressure they face in competition and success is well-known and can be compounded by adverse events in life (23). Such an adverse event of the coronavirus pandemic made professional athletes especially vulnerable to mental illness with possible implications for general health. Indeed, research reports indicate that athletes are reaching out for mental health support, highlighting pressures of boredom and stress related to the social isolation they are forced to endure. Generally, there was a high prevalence of mental health issues and psychosocial challenges among professional athletes during the coronavirus pandemic. These studies have shown that professional athletes are not immune to this stress and negatively affected. The isolation from their athletic team, reduced activity and training, lack of formal coaching, and lack of social support from fans and media have caused emotional distress in athletes (6).

The evaluated investigations included stressors such as fear of being infected, lack of access to training facilities, lack of ability to continue practicing their skills, canceled matches, lack of social support, and lack of or reduced income. These stressors may lead to outcomes such as disturbed sleep, eating disorders, obsessive-compulsive disorders, family conflicts, and unhealthy coping mechanisms such as smoking and increased alcohol intake. The COVID-19 pandemic impacting the training schedules of athletes has affected their sleeping habits and caused unhealthy habits and coping mechanisms such as increasing their carbohydrate intake and preferring sedentary behavior above active behavior (15). This evidence shows that the effects of lockdown are more severe and multifaceted than just a scheduled absence from training activities.

Another issue that could occur as a result of the constraints imposed is of a financial nature. Competitions, matches, and leagues were canceled due to the outbreak, and this could be the only source of income for certain athletes. While professional athletes in leading leagues do not face the same financial restrictions, cancelations of many sporting events worldwide would impact many teams globally (24). This presents another specific range of concerns, as financial hardship may lead to more psychological distress and, in addition, a decreased income may lead to an inability to afford nutritious food, athletic equipment, or access to training facilities. This may further raise the risk of reduced psychosocial well-being, mental health and well-being, and cardiovascular health.

These outcomes further have consequences on general and cardiovascular health, causing a vicious cycle of events. The potential inability of these athletes to manage their stress and coping mechanisms may lead to long-term depression and other detrimental health effects (25). Although it is difficult to accurately forecast COVID-19's psychological and emotional impact in the current unprecedented situation, we can expect that it can serve as a negative stressor for many athletes.

Mental health cannot be separated from physical health, as mental health symptoms and associated systemic disorders increase the risk of physical injury and delay recovery. Physical activity has beneficial effects for both the prevention and treatment of different diseases and psychiatric diseases such as depressive and anxiety disorders (26). Psychological stress and physical activity have opposite effects on parameters that affect cardiovascular status (27). Even with physical training, cardiovascular disease and risk factors are fairly prevalent in athletes (28). In terms of COVID, having preexisting conditions may lead to worse outcomes, and therefore this is an important aspect to consider when planning the safe return of athletes. Guidelines need to be developed for the diagnosis and management of mental health symptoms and disorders in elite athletes (29). Cardiorespiratory rehabilitation strategies are essential for a return-to-play with sufficient cardiorespiratory fitness and reduced psychosocial stress in elite athletes after COVID-19 infection (30). Wilson et al. (30) highlight the need to have safe guidelines for athletes to return to normal sporting activities. It also shows how the long-term impact of the disease itself can hinder athletes from returning to normal activities. This would ultimately add to the mental impact of COVID-19 on athletes.

### Strengths and Limitations

In the context of the COVID-19 pandemic and professional athletes, the strengths of this analysis include its exploration of peer-reviewed articles. Its drawbacks must, however, also be recognized. In accordance with the scoping analysis procedure, the included articles were not evaluated for accuracy. Study bias may also have been introduced by removing gray literature and non-English language texts. Future studies would benefit from a researching a broader range of bibliographic databases.

## Conclusion

As exposed in this scoping review, several studies on the impact of the coronavirus pandemic on athletes indicate that this pandemic may act as a triggering life event that would increase occupational stress to afflict mental health and well-being. Afflicted mental health and well-being could also impact their cardiovascular health, which in turn could adversely impact further their mental health. Research on mental and cardiovascular health shall further investigate the identified risk effects brought on from a combination of stressors directly associated with COVID-19 exposure, isolation, lack of physical exercise, reduced income, and fear of unemployment in athletes. Such stressors and their impact on athletes' healthcare should be tackled by psychosocial aid and occupational therapy programs to better deal with the coronavirus pandemic.

## Author Contributions

OB and LC conceptualized the review and initiated the draft of the manuscript. RH, MA, and DS screened the articles, contributed to the abstraction, and reviewed the manuscript. HA and KD contributed to the abstraction and validation of data and preparation of the manuscript. All authors critically reviewed and revised the final version of the manuscript and contributed significantly to the conceptualization and reporting of the review.

## Conflict of Interest

The authors declare that the research was conducted in the absence of any commercial or financial relationships that could be construed as a potential conflict of interest.

## Abbreviations

DASS-21: Depression-Anxiety-Stress Scale-21
IES-R: Impact of Events Scale-Revised
IPAQ-SF: International Physical Activity Questionnaire Short Form
AIMS: Athletic Identity Measurement Scale
CERQ: Cognitive Emotion Regulation Questionnaire
GAD-7: General Anxiety Disorder-7 Item
PHQ-9: Patient Health Questionnaire-9 Item
PFABS: Hospital for Special Surgery Pediatric Functional Activity Brief Scale
PedsQL: Pediatric Quality of Life Inventory 4.0
AIMS: Athletic Identity Measurement Scale
SSB: Socially Supportive Behaviors
MHC-SF: Mental Health Continuum Short Form instrument
PROMIS: Patient-Reported Outcomes Measurement Information System
POMS: Profile of Mood States, Spanish validated version
WLEIS-S: Wong Law Emotional Intelligence Scale Short form, Spanish validated version
BRS-II: Brief Resilience Scale, Spanish validated version.
